# Protocol for the Process Evaluation of the SENSE‐Cog Sensory Support Intervention Field Trial to Improve Quality of Life for Older People Receiving Home Care in Australia

**DOI:** 10.1111/hex.70305

**Published:** 2025-05-25

**Authors:** Helen Gurteen, Melinda Toomey, Bronwyn Franco, Rebecca Bennett, Dayna R. Cenin, Najwan El‐Saifi, Melanie Ferguson, Yuanyuan Gu, Chyrisse Heine, Lisa Keay, Sheela Kumaran, Sabrina Lenzen, Iracema Leroi, Judy A. Lowthian, Carly J. Meyer, Leander K. Mitchell, John Newall, Nancy A. Pachana, Marianne Piano, Smriti Raichand, Emma Scanlan, Hamid R. Sohrabi, Piers Dawes

**Affiliations:** ^1^ Centre for Hearing Research, School of Health and Rehabilitation Sciences University of Queensland Brisbane Australia; ^2^ National Acoustic Laboratories Sydney Australia; ^3^ School of Population and Global Health The University of Western Australia Perth Australia; ^4^ School of Allied Health Curtin University Perth Australia; ^5^ Macquarie University Centre for the Health Economy, Macquarie Business School & Australian Institute of Health Innovation Macquarie University Sydney Australia; ^6^ Institute of Health and Wellbeing Federation University Australia Ballarat Australia; ^7^ School of Optometry and Vision Science UNSW Sydney Sydney Australia; ^8^ Centre for the Business and Economics of Health The University of Queensland Brisbane Australia; ^9^ Global Brain Health Institute, Department of Psychiatry, School of Medicine Trinity College Dublin Dublin Ireland; ^10^ Bolton Clarke Research Institute Brisbane Australia; ^11^ School of Psychology The University of Queensland Brisbane Australia; ^12^ Department of Linguistics, Faculty of Medicine, Health and Human Sciences, The Australian Hearing Hub Macquarie University Sydney Australia; ^13^ Department of Optometry and Vision Sciences, Melbourne School of Health Sciences University of Melbourne Melbourne Australia; ^14^ National Vision Research Institute Australian College of Optometry Melbourne Australia; ^15^ National Centre for Healthy Ageing Monash University Melbourne Australia; ^16^ Hearing Australia Sydney Australia; ^17^ Centre for Healthy Ageing, Health Futures Institute Murdoch University Perth Australia

**Keywords:** dementia, hearing, home care, older adults, process evaluation, vision

## Abstract

**Background:**

A field trial of a home‐delivered hearing and vision support intervention will assess its impact on the quality of life and well‐being of home care recipients with hearing and/or vision impairment and their care partners.

**Aims:**

This paper outlines the protocol for a process evaluation of the field trial. The process evaluation aims to identify discrepancies between expected and actual outcomes, understand contextual influences, assess implementation fidelity and evaluate the feasibility, appropriateness and acceptability of the intervention.

**Methods:**

Data will be collected from 87 home care recipients with hearing/vision impairment, their care partner and the sensory therapist who will deliver the intervention at multiple points during the 3‐month intervention. Likert‐scale ratings for feasibility, appropriateness and acceptability will be gathered. Proxy measures of fidelity, such as intervention session completion rates, will be obtained to ascertain whether the intervention was delivered as designed. Post‐intervention, 20% of participants will complete semi‐structured interviews to explore contextual and causal factors. Data analysis will include descriptive statistics, regression analysis and thematic qualitative analysis.

**Discussion:**

The process evaluation will elicit the perspectives of home care recipients and their care partners regarding the intervention experience.

**Patient or Public Contribution:**

Older adults with lived experience with dementia and hearing and/or vision will contribute to the proposed research by shaping the interview topic guide to ensure its appropriateness and relevance for the target population. Their insights will result in a more rigorous study and improve the likelihood of the final intervention meeting real‐world needs.

## 1 Introduction

As populations age, promoting quality of life for older adults becomes more pressing [1]. Both hearing and vision impairment negatively impact quality of life, social engagement and mental health, with hearing and/or vision impairments experienced by an estimated 70% of people aged over 70 years [2, 3, 4]. Around one‐third of older people may also be living with mild cognitive impairment (MCI) or dementia [5]. Older adults with comorbid cognitive and hearing/vision impairments are less likely to have hearing/vision needs identified and impairments addressed than individuals without cognitive impairments [6], exacerbating the impact of cognitive impairment on quality of life, functional ability and communication [7].

A home‐delivered hearing and vision support intervention (SENSE‐Cog Sensory Support Intervention [SSI]) was co‐developed with adults living with dementia and their care partners in Europe. The SENSE‐Cog intervention was trialled across Europe to evaluate its efficacy in improving the quality of life amongst people with dementia and hearing/vision impairment, by addressing their hearing and/or vision needs [8]. Immediately following the intervention, quality of life was found to have significantly improved, but these improvements were not maintained at 36 weeks post‐intervention, indicating a loss of the intervention's initial benefits [8, 9]. Despite the lack of continued improvement in this population, the initially encouraging result suggests there could be benefits in relation to home care settings in Australia.

Within the Australian setting, home‐based care is provided to older adults through the Australian government's My Aged Care programme [10]. The level of care provided is determined based on need, and services can include personal care, nursing, allied health services, domestic assistance, meal provision, transportation and social support. In accordance with a consumer‐directed‐care model, recipients select their service providers to suit their own needs [10]. This differs substantially from the context in which the SENSE‐Cog intervention was previously trialled, where participants were community‐dwelling people with dementia, not all of whom received formal care services.

We recently worked with home care service users, hearing/vision clinicians and home care providers to adapt the SENSE‐Cog SSI for Australian home care settings [11]. A complex individualised intervention, the adapted SSI, incorporates eight components including the identification of hearing or vision impairment, optimisation of hearing and vision functioning, hearing/vision assistive device training, home‐based functional assessment, communication training, health and social services support, connecting with others and interests, and environmental modifications and assistive devices. A few key modifications to the intervention included adaptation of materials to an online learning environment to allow access to materials between intervention sessions, expansion of training to include assistive technology and phone‐based apps, and collation of local resources that highlight available community groups, specialist services and referral materials [11]. These modifications were made to align the intervention with the care needs identified by Australian home care recipients and to address the gaps highlighted by professionals working in the Australian setting [11, 12].

The SSI (or components of the SSI) are intended to be offered as part of home‐based aged care packages—which are currently available to adults in Australia who are aged over 65 years and need coordinated care to stay in their own homes. The SSI is designed to be delivered over a 3‐month period by a sensory therapist—a person with a background in allied health—in the home of the older adult. Based on qualitative research demonstrating the importance of involving caregivers in interventions [13], the SSI was designed to include the involvement of a care partner (e.g., partner or son or daughter), with the secondary goal of addressing the impact of sensory impairment on care partners.

A pre‐post mixed‐methods field trial of the adapted SSI has been designed to determine the effectiveness and cost‐effectiveness of the intervention for older adults with hearing and/or vision impairment receiving home care services in Australia. The protocol for the trial was reported in detail in Toomey et al. [12]. Briefly, the trial aims to evaluate: (i) the impact of the adapted intervention on the quality of life, well‐being, functional ability, behaviours and sensory environment of homecare recipients; (ii) the impact on care partner's quality of life, well‐being and their relationship with the homecare recipients and (iii) the costs associated with the intervention and its cost‐effectiveness.

In addition to evaluating the outcomes of the intervention, there is a need to consider the role of implementation‐related factors, mechanisms of impact and context in the success or failure of complex interventions [14]. Conducting a process evaluation allows researchers to ascertain whether any success or failure could be attributed to the intervention itself rather than to failures in implementation [14]. Process evaluation not only leads to an understanding of cause and effect but is also integral to clinical implementation of an intervention [15].

In accordance with the UK Medical Research Council's (UK MRC) guidance on process evaluation, the feasibility, appropriateness, fidelity and acceptability of complex interventions should be evaluated alongside an effectiveness and implementation trial [14]. Feasibility is defined as the degree to which an intervention is effectively implemented in a specific environment [16]. Appropriateness refers to how well the intervention aligns with and is relevant to those implementing or benefiting from it [16]. Fidelity examines the extent to which the intervention is delivered as intended [16]. Acceptability is the degree to which the intervention is perceived as agreeable, palatable or satisfactory to the intervention recipients or administrators [16].

The adapted SSI comprises multiple components, which are intended to be delivered in a flexible manner depending on the needs and preferences of the individual. The study will likely include individuals who have different levels of support at home (e.g., live with a partner or live alone), have different levels of cognitive function and hearing/vision impairment, and include home environments that may differ in ways that are relevant to the hearing/vision intervention (e.g., lighting levels or background noise). A process evaluation is critical to identifying all potential sources of variation, such as those highlighted above, and determining which have an impact on the outcomes of the intervention.

This paper describes the protocol for the process evaluation of the field trial of the SENSE‐Cog SSI adapted for older adults with hearing and/or vision impairments in Australian home care settings. The process evaluation outlined here aims to explore potential discrepancies between expected and observed outcomes and to understand how context may influence trial outcomes. It also aims to determine the extent to which the intervention was implemented as intended and to identify any contextual issues that influenced intervention delivery, alongside causal mechanisms through which the intervention achieved or did not achieve impact. In this way, the feasibility, appropriateness and acceptability of the intervention for the participants, and the sensory therapist who delivered the intervention, will be determined.

## 2 Materials and Methods

**Figure 1:**
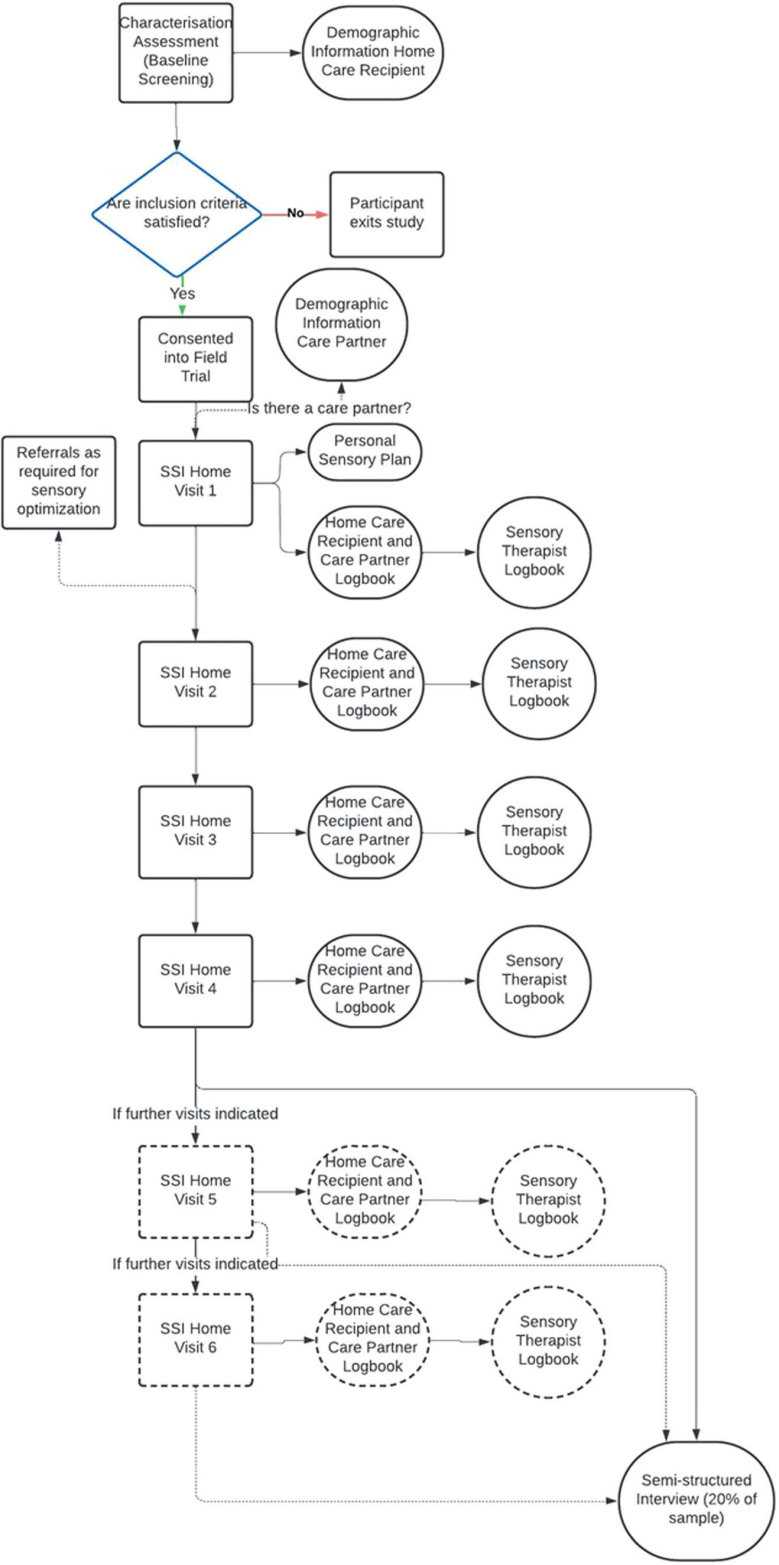
Flow chart of process evaluation data collection points related to field trial implementation. Process evaluation data are shown in circle or oval shapes, with the field trial structure shown as rectangles.

A mixed‐methods design will be utilised to allow for an in‐depth exploration of the implementation of the intervention. Following the UK MRC's guidance for process evaluation of complex interventions, the design will take into consideration the need for differentiation between the outcome evaluation and the process evaluation to prevent undue influence of one on the other [14]. Process evaluation data will be analysed independently of the analysis of the field trial outcome data, with analysis of the process evaluation data led by a separate member of the research team. Data collection for process evaluation measures will occur at multiple points throughout the intervention (Figure 1).

### 2.1 Setting

As outlined in the field trial protocol, the research will be conducted in metropolitan settings in South East Queensland [12]. The trial will be conducted in participants' homes, which may include retirement villages or community‐based private residences. This approach ensures that the study is carried out in a familiar and comfortable environment for the participants and mimics the settings in which the intervention would be delivered clinically should it be implemented by home care providers.

### 2.2 Participants and Sample Size

To ensure comprehensiveness, all 87 participants in the field trial will participate in the process evaluation. The inclusion and exclusion criteria for home care recipients and for care partners to enter the field trial are outlined below.

#### 2.2.1 Home Care Recipients Inclusion Criteria

People aged 65 years or older will be eligible to participate if they: (i) have cognitive function ranging from normal to moderately advanced dementia (Stage 6, as per the 7‐stage Functional Assessment Staging Tool Score [17]), (ii) are living in their own home and receiving home care services; (iii) can provide informed consent or can assent and have a care partner who can provide proxy consent, (iv) have adult‐acquired hearing and/or vision impairment (hearing impairment thresholds worse than 20 dB HL at 1000 Hz or 2000 Hz, or worse than 35 dB HL at 4000 Hz, or 8000 Hz in the better ear as measured with the HearX HearTest [18]; monocular visual acuity no better than 6/12 in the better eye as measure with the Peek Acuity App [19]). People will be ineligible to participate if they have pre‐arranged cataract(s) surgery within the intervention period.

#### 2.2.2 Care Partner Inclusion Criteria

Care partners of a person aged 65 years and older receiving home care services may choose to participate in the project provided they are: (i) aged 18 years or older, (ii) are an informal caregiver of a home care recipient participating in the study, (iii) are able to provide consent and (iv) can converse in English. Care partners who have less than weekly contact with the person receiving home care services will not be eligible to take part.

### 2.3 Ethical Considerations

This study has received approval from The University of Queensland Human Research Ethics Committee (2023/HE002236). As the process evaluation forms part of the same research project as the field trial, participants will be asked to consent to the one project. Participants will be provided with an information sheet about the study, will discuss it with a researcher and give written consent, with the freedom to withdraw at any time. Semi‐structured interviews will be audio recorded with the participant's consent. For participants with dementia, accessible materials and plain language explanations will be available, along with proxy consent from legal guardians and assent from the individual with dementia. If participants are unable to sign consent forms, a designated person can complete the forms in their presence, with the researcher countersigning. For those unable to read information sheets and consent forms, documents will be read aloud to participants for verbal agreement.

### 2.4 Intervention Description and Theoretical Basis

A full description of the SSI is provided as part of the field‐trial protocol [12]. A brief overview is provided here to provide context for the implementation evaluation. The intervention was developed in accordance with the UK's MRC guidelines for the development and testing of complex interventions [20]. The original European SENSE‐Cog intervention was co‐developed with people living with dementia and their care partners [21]. The intervention was designed to be individualised according to each person's hearing/vision needs and preferences and to be delivered in the person's own home.

With input from Australian home care recipients, hearing/vision clinicians and home care providers, the SENSE‐Cog intervention was adapted to meet the hearing/vision needs of people with hearing/vision impairment in Australian home care settings. The adapted intervention includes eight components: (a) identification of hearing/vision impairment (i.e., hearing and vision assessment; questionnaire about sensory needs and preferences), (b) optimisation of hearing and vision function (e.g., provision of glasses and hearing aids), (c) sensory device training (e.g., use and maintenance of hearing aids and glasses), (d) home‐based functional assessment (e.g., evaluation of the home environment for factors that may impact functionality such as lighting and reverberation), (e) communication training (e.g., for a person with hearing loss, getting their attention before speaking to them or for visual impairment making written communication big and bold), (f) referral to any relevant health, mental health and social services, (g) connecting with others (e.g., involvement with local social or interest groups) and (h) environmental modifications and assistive devices (e.g., magnifiers or television streamers) [11]. All participants will receive elements of Components A–E. The provision of Components F–H is only intended to be administered in cases where they are expected to address the identified needs of the individual home care recipient.

The intervention will be administered by a ‘sensory therapist’—a person with a background in allied health (e.g., social work, occupational therapy, audiology and optometry) and a basic level of training in screening for hearing and vision function and delivering the intervention. Training for the sensory therapist will be provided via both online learning materials developed by audiology, optometry and healthy ageing experts as well as via face‐to‐face training in screening protocols delivered by qualified optometrists and audiologists [11]. The intervention will be delivered in the participant's own home across 4–6 visits, over a 3‐month period.

### 2.5 Process Evaluation Measures

The sensory therapist delivering the intervention will play a central role in process evaluation data collection during the intervention field trial. Logbooks for each intervention visit will be completed online by participants and care partners using Qualtrics (Qualtrics XM, Seattle, Washington State) and will capture visit details, sensory support goals and actions, and survey data. The logbook includes questionnaires with Likert‐style ratings to assess various implementation factors. These include feasibility, appropriateness, acceptability, recipient engagement with the intervention as rated by the care partner when a care partner is enrolled in the study, adaptation to sensory devices (e.g., hearing aids), and how confident the care partners feel in supporting the homecare recipient in using any hearing/vision device(s).

The sensory therapist will assist the home care recipient and care partner in filling in their logbooks together at the end of each intervention visit. It is anticipated that it will take 10 min per visit to complete the logbooks. Participants will have the option for the questionnaires to be administered as a face‐to‐face structured interview. This flexible methodology aims to accommodate the diverse needs and preferences of all participants, allowing for varying levels of digital literacy, vision and cognitive ability.

The sensory therapist will complete the sensory therapist logbook using Qualtrics on completion of each intervention visit. Likert‐style ratings for feasibility, appropriateness, acceptability and recipient engagement will again be utilised. Furthermore, the sensory therapist will record qualitative notes concerning their experiences, ideas and personal reactions to delivering the intervention, as well as make observations about the uptake and use of the intervention by participants.

Semi‐structured interviews will be conducted with 20% of home care recipients and care partner participants by a separate member of the research team following completion of the intervention. Purposive sampling will be used for the semi‐structured interviews to maximise diversity of impairment, cognitive function and intervention components experienced. Semi‐structured interviews may be conducted via Zoom audio calls (Zoom Video Communications Inc., San Jose, California), telephone or face‐to‐face in the participant's home according to their preference. The interview topic guide will explore factors including acceptability of the intervention, contextual issues, and barriers and enablers to intervention implementation (see Appendix A). This guide will be piloted and reviewed by a patient and public involvement advisory group to ensure that the questions are appropriate and easy to understand. To support the participation of people with dementia, open‐ended interview questions may be rephrased to be more structured, and care partners will be included in the semi‐structured interview to support the person with dementia. Each interview is expected to last about 1 h and will be recorded and transcribed verbatim for systematic qualitative analysis. The interviewer will take notes regarding nonverbal communication throughout the semi‐structured interviews to allow for a deeper appreciation of the participant's interactions.

The data collection tools outlined above allow for the derivation of several different implementation outcome measures. The outcome measures selected were informed by the UK's MRC guidelines on the development and testing of complex interventions and Proctor et al.'s taxonomy of implementation outcomes [16, 20]. The six implementation outcomes selected for investigation are feasibility, appropriateness, fidelity, acceptability, mechanisms of change and context. An overview of how the data collected directly relates to these implementation measures is provided in Table 1.

**Table 1:** Implementation outcomes assessed as related to the data collection tools.

Implementation outcome	Data collection tools	Data collected from and about	Data collection time point	Methodology utilised
Feasibility	SSI—Participant and Support Person Logbook	Sensory therapist about home care recipient and care partner, where applicable	Upon completion of each home visit session	Quantitative
	SSI—Sensory Therapist Logbook	Sensory therapist about home care recipient and self	Upon completion of each home visit session	Quantitative and qualitative
Appropriateness	SSI—Participant and Support Person Logbook	Sensory therapist about home care recipient and care partner, where applicable	Upon completion of each home visit session	Quantitative
	SSI—Sensory Therapist Logbook	Sensory therapist about home care recipient and self	Upon completion of each home visit session	Quantitative and qualitative
Fidelity	SSI—Sensory Therapist Logbook	Sensory therapist about intervention delivery	Upon completion of each home visit session	Quantitative
Acceptability	SSI—Participant and Support Person Logbook	Sensory therapist about home care recipient and care partner, where applicable	Upon completion of each home visit session	Quantitative
	SSI—Sensory Therapist Logbook	Sensory therapist about home care recipient and self	Upon completion of each home visit session	Quantitative and qualitative
	Semi‐structured Interviews	Researcher about home care recipients and care partners	Immediately post‐intervention	Qualitative
Context	Home Care Recipient Demographic Information	Researcher about home care recipients	Baseline screening	Quantitative
	Care Partner Demographic Information	Sensory therapist about the care partner	Before or at the initial home visit	Quantitative
	SSI—Sensory Therapist Logbook	Sensory therapist about home care recipient and self	Upon completion of each home visit session	Quantitative and qualitative
	Semi‐structured Interviews	Researcher about home care recipients and care partners	Immediately post‐intervention	Qualitative
Causal mechanisms	Semi‐structured Interviews	Researcher about home care recipients and care partners	Immediately post‐intervention	Qualitative

In line with Proctor et al.'s definition, feasibility is conceptualised as the degree to which an intervention is effectively implemented in a specific environment [16]. Feasibility will be measured via the validated Feasibility of Intervention Measure (FIM), a 4‐item questionnaire rated on a 5‐point Likert scale (see Table 2) [22]. Appropriateness refers to how well the intervention aligns with and is relevant to those implementing or benefiting from it [16]. The validated Intervention Appropriateness Measure (IAM), a 4‐item questionnaire, will be utilised and is also rated on a 5‐point Likert scale (see Table 2) [22].

**Table 2:** Process evaluation outcome measures that assess feasibility, appropriateness, (Feasibility Intervention Measure and Intervention Appropriateness Measure questionnaires [22]. Adapted from Weiner et al.) and acceptability (Theoretical Framework of Acceptability from Sekhon et al.) under Creative Commons Attribution CC BY 4.0 https://creativecommons.org/licenses/by/4.0/.

Implementation factor	Question	Response scale
Feasibility Intervention Measure (FIM)	1. The sensory support strategy/action seems implementable.	Strongly agree (5) to strongly disagree (1)
	2. The sensory support strategy/action seems possible.	
	3. The sensory support strategy/action seems doable.	
	4. The sensory support strategy/action seems easy to do.	
Intervention Appropriateness Measure (IAM)	1. The sensory support strategy/action seems fitting for me.	Strongly agree (5) to strongly disagree (1)
	2. The sensory support strategy/action seems suitable for me.	
	3. The sensory support strategy/action seems applicable to me.	
	4. The sensory support strategy/action seems like a good match for me.	
Theoretical Framework of Acceptability (TFA)	Affective attitude 1a. How comfortable do you feel using your hearing device? 1b. How comfortable do you feel using your vision device?	Very comfortable (4) to very uncomfortable (0)
	Burden 2a. How much effort did it take to use your hearing device? 2b. How much effort did it take to use your vision device?	Very little effort (4) to huge effort (0
	Ethicality 3. How fair is using a sensory support device to people with dementia?	Very fair (4) to very unfair (0)
	Perceived effectiveness 4a. The hearing device has improved my activities of daily living. 4b. The vision device has improved my activities of daily living.	Strongly agree (4) to strongly disagree (0)
	Intervention coherence 5. It is clear to me how the sensory support intervention will help me with activities of daily living.	Strongly agree (4) to strongly disagree (0)
	Self‐efficacy 6. How confident did you feel about engaging with the sensory support intervention activities and materials?	Very confident (4) to very unconfident (0)
	Opportunity costs 7. The sensory support intervention interfered with my other priorities.	Never (4) to very frequently (0)
	General acceptability 8. How acceptable was the sensory support intervention to you?	Completely acceptable (4) to completely unacceptable (0)

Acceptability is the degree to which the intervention is perceived as agreeable, palatable or satisfactory to the intervention recipients or administrators [16]. Exploration of acceptability will be guided by the seven constructs of the Theoretical Framework of Acceptability for Healthcare Interventions (TFA) [23]. The TFA is an established framework for assessing the acceptability of interventions where acceptability is conceived to be multifaceted and comprised seven constructs: affective attitude, burden, ethicality, opportunity costs, perceived effectiveness and self‐efficacy [23]. The generic TFA questionnaire was adapted to create 11 questions [16]. Further qualitative information about the acceptability of the SSI will be gathered via the semi‐structured interviews, which will be conducted upon completion of the intervention.

Fidelity will be examined to ascertain the extent to which the intervention is delivered as intended [16]. Consistent with UK MRC recommendations, fidelity will be determined via proxy measures [14] to assess adherence, dosage, quality of intervention delivery, participant responsiveness and programme differentiation [24]. Proxy measures include: intervention visit completion rates, duration of each intervention session, the support offered—including type of corrective devices, the environmental modifications to support sensory function made, the number and types of referrals to extra‐services or social opportunities, the specific resources engaged with, and generalised feedback. These data will be collected via the sensory therapist logbook.

Context is the set of circumstances surrounding an intervention that encompasses intervening variables such as the physical, social, cultural and political environments that interact with and influence an intervention's implementation [25]. Exploration of context will be guided by the Context and Implementation of Complex Interventions (CICI) Framework that assists in the structural analysis of implementation context at individual (e.g., individual beliefs, attitudes and skills), meso (e.g., community norms, social networks or organisational resources) and macro levels (e.g., societal values and national policies) [25].

**Figure 2:**
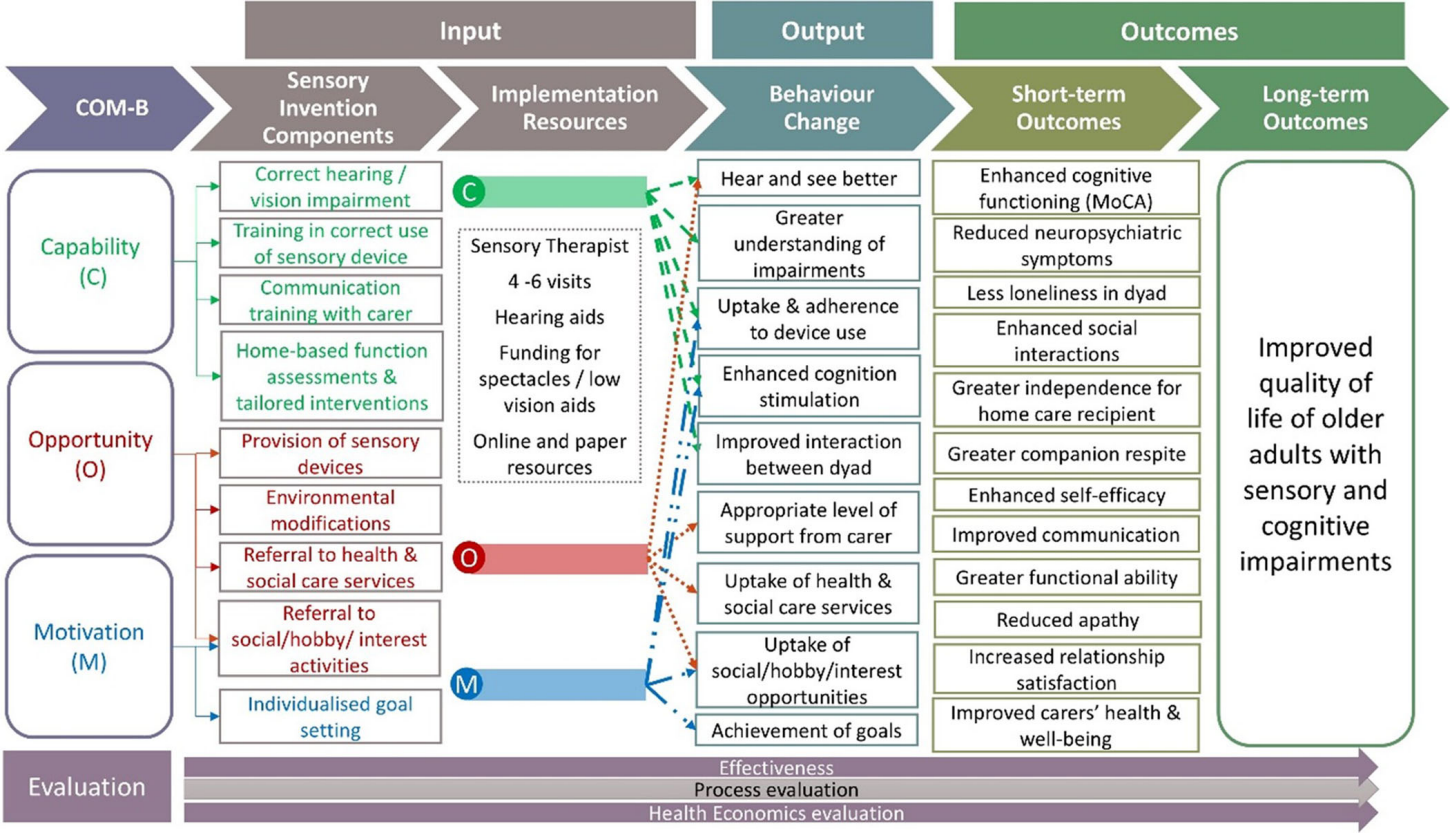
Logic model for Sensory Support Intervention showing how the domains of the ‘COM‐B Behaviour Change Wheel’ and the components of the intervention link together and the expected behaviour changes, short‐term outcomes and long‐term outcomes.

The study will also investigate causal mechanisms of any improvement, or lack of improvement, elicited by the field trial. The investigation of these causal mechanisms will be guided by the Behaviour Change Wheel and COM‐B model [16]. This model posits that behaviour is the result of the interplay between a person's capability (C) (their knowledge and skills), opportunity (O) (their environment) and motivation (M) (their conscious and unconscious decision processes) [26]. Figure 2 illustrates the relationship between the project's needs mapped to the COM‐B model, inputs, activities and outcomes, demonstrating how resources and actions are anticipated to lead to results. Pathways and mechanisms will be explored during semi‐structured interviews that will take place after the intervention.

### 2.6 Data Management Plan

All data will be securely stored on The University of Queensland's Research Data Management System to ensure confidentiality. During transcription of the semi‐structured interviews, all identifying data will be redacted with names replaced with unique identifiers. An encrypted protected spreadsheet will be utilised to allow for unique identifiers to be linked to participant names during the delivery of the intervention. This document will be deleted upon project completion. With the consent of participants, non‐identifiable data will be archived in a publicly accessible repository, in accordance with the National Health and Medical Research Council's Open Access Policy (2022) and Findable, Accessible, Interoperable and Reusable (FAIR) Principles [27].

### 2.7 Patient and Public Involvement

The proposed process evaluation will include input from a Patient and Public Involvement (PPI) advisory group. The PPI advisory group members will have direct experience caring for someone with dementia, hearing and/or vision impairment, or will be living with dementia, hearing and/or vision impairment themselves. PPI input has been reported to improve the relevance of research to the target population [28].

PPI input will be reported according to Staniszewska and colleagues' Guidance for Reporting Involvement of Patients and the Public‐2 (GRIPP‐2), which was designed to enhance transparency and consistency of PPI reporting in research [28]. The GRIPP2‐short form checklist [28] will be utilised to capture the aims, methods, results, outcomes and critical perspectives gained from PPI.

### 2.8 Data Analysis

The implementation evaluation will answer two key research questions. Firstly, to what extent was the intervention implemented as intended and what were the contextual issues that influenced intervention delivery and causal mechanisms through which the intervention achieved or did not achieve impact? Secondly, is the intervention feasible, appropriate and acceptable to participants?

To address the first research question, descriptive statistics, including frequency and duration analysis, will be generated for the proxy measures of fidelity. Component utilisation analysis will be used to identify patterns in the distribution of components across participants. Regression analysis will also be undertaken to examine the relationships between different fidelity measures and contextual issues to determine contextual predictors of protocol adherence.

Data from semi‐structured interviews will be transcribed into NVivo (Lumivero, Denver, Colorado) [29] and independently coded by two members of the research team, neither of whom will be the sensory therapist (H.G. and M.T.). Both inductive and deductive approaches will be employed in the thematic analysis of the material to achieve a more robust and nuanced understanding of the topic. This analysis will adhere to Braun and Clarke's method, which includes the following steps: data familiarisation, generating initial codes, searching for themes, reviewing themes, defining themes and writing up [30]. The iterative development of themes from semi‐structured interviews will allow for more in‐depth exploration of the themes spotted in the early transcripts in later transcripts, providing a more comprehensive analysis of the data. Contextual issues that influenced intervention delivery and causal mechanisms through which the intervention achieved or did not achieve impact will be investigated using content analytic approaches.

Feasibility, appropriateness and acceptability will be examined quantitatively via descriptive statistics from the perspective of both the participants and the sensory therapist. More in‐depth exploration of the participant's insights into these implementation factors will be undertaken through the qualitative analysis of the semi‐structured interviews as described above.

## 3 Discussion

The inclusion of a comprehensive process evaluation of the SENSE‐Cog SSI field trial for Australian home care recipients will increase the understanding of cause and effect beyond that provided by an outcome evaluation alone. Process evaluation considers the role of implementation, mechanisms of impact, context and their contribution to the outcome achieved [14]. Without consideration of these factors, it would not be possible to ascertain to what extent any improvement seen post‐intervention could be causally attributed to the intervention itself or to what extent the intervention is feasible, appropriate and acceptable.

The process evaluation protocol outlined in this paper has several strengths. The collection of quantitative evaluation outcome measures from all participants in the field trial ensures complete capture of all data relating to the implementation of the intervention. Collection of qualitative data from a subset of the participants complements quantitative data and allows for an in‐depth understanding of causal and contextual factors that may affect the outcomes of the intervention. Embedding the process evaluation measures throughout the field trial provides an opportunity for the identification of changes in contextual factors during the course of the intervention [27].

The overlap in personnel involved in both the field trial and the process evaluation is a limitation of the current design. The sensory therapist will deliver the intervention and administer the process evaluation measures that are collected during the intervention period. This could potentially lead to biased reporting of participants' views on feasibility, acceptability and appropriateness due to participants' reluctance to criticise [14]. To minimise this risk, participants will be offered the option of completing the logbooks independently if they prefer. Information will be collected regarding whether participants opt to complete the logbooks independently, and this will be considered during analysis. Additionally, the semi‐structured interviews will be conducted by members of the research team who are not directly involved in the delivery of the intervention to the participants.

Insights from the process evaluation would enable refinement of the intervention to better meet the hearing and vision care needs of older Australians. Insights gained from this process evaluation will also guide future implementation of the SSI. The entire intervention, or components of the intervention, could be integrated within home care packages in Australia that are customisable according to individual needs. Data on intervention fidelity and causal mechanisms will provide evidence on the core components of hearing and vision support packages that could be offered in home care packages [31]. Understanding contextual issues that affect the delivery and effectiveness of the intervention could also inform future adaptation of the intervention into home care packages [25].

## 4 Conclusion

In conclusion, the proposed process evaluation will generate insights into the causal and contextual factors that impact the delivery and effectiveness of the SENSE‐Cog SSI. This process evaluation will ensure that the views of home care recipients and their care partners are represented in the context of the findings and outcomes of this study, a perspective that is vitally important if the intervention is to improve the quality of home care and promote good quality of life among older Australians.

## Author Contributions

**Helen Gurteen:** writing – original draft, methodology, writing – review and editing, formal analysis. **Melinda Toomey:** formal analysis, methodology, writing – review and editing, project administration. **Bronwyn Franco:** writing – review and editing, methodology. **Rebecca Bennett:** methodology, writing – review and editing, funding acquisition. **Dayna R. Cenin:** methodology, writing – review and editing. **Najwan El‐Saifi:** methodology, writing – review and editing. **Melanie Ferguson:** methodology, writing – review and editing. **Yuanyuan Gu:** methodology, writing – review and editing, funding acquisition. **Chyrisse Heine:** funding acquisition, methodology, writing – review and editing. **Lisa Keay:** funding acquisition, methodology, writing – review and editing. **Sheela Kumaran:** funding acquisition, methodology, writing – review and editing. **Sabrina Lenzen:** funding acquisition, methodology, writing – review and editing. **Iracema Leroi:** conceptualisation, funding acquisition, writing – review and editing, methodology. **Judy A. Lowthian:** methodology, writing – review and editing, funding acquisition. **Carly J. Meyer:** funding acquisition, methodology, writing – review and editing. **Leander K. Mitchell:** funding acquisition, methodology, writing – review and editing. **John Newall:** funding acquisition, methodology, writing – review and editing. **Nancy A. Pachana:** funding acquisition, methodology, writing – review and editing. **Marianne Piano:** funding acquisition, methodology, writing – review and editing. **Smriti Raichand:** methodology, writing – review and editing. **Emma Scanlan:** funding acquisition, methodology, writing – review and editing. **Hamid R. Sohrabi:** funding acquisition, methodology, writing – review and editing. **Piers Dawes:** conceptualisation, funding acquisition, methodology, writing – review and editing, supervision.

## Ethics Statement

This study protocol was reviewed and approved by The University of Queensland Human Research Ethics Committee, approval number 2023/HE002236.

## Consent

Written consent will be obtained from participants or from their proxy (e.g., people with dementia). Assent will be gained from people with dementia.

## Conflicts of Interest

The authors have no conflicts of interest to declare. H.R.S. reports being a Director of SMarT Minds WA, Australia. H.R.S. has had or is receiving personal reimbursements or research support from the Pharmaceutical and Nutraceutical companies including Alector, Alnylam Pharmaceuticals, CWEK Pty Ltd and Biogen pharmaceuticals.

## Data Availability

The de‐identified datasets generated and/or analysed during this study will be made publicly available via the University of Queensland Research Data Manager (UQ purposes RDM).

## Appendix 5

**Topic Guide**

***Semi‐structured Interviews With Home Care Recipients and Family Members***

**Effectiveness Questions**

**Table:**

1	Over the last few months, the sensory therapist has visited you several times. How did you find those visits?
2	From your perspective, what was the most useful component of the intervention? What was the least?
3	How has the sensory support intervention helped you with your activities of daily life and communication with your key person?
4	Have you noticed any lasting effects of the intervention on your overall well‐being, cognitive function or quality of life?
5	Would you like to see this support continued? Tell me more.

**Process Evaluation Questions**

***Acceptability***

**Table:**

1	Affective attitude	How do you feel about the sensory support intervention? What did you like? What did you dislike?
2	Burden	How easy or difficult was it to complete the different components of the sensory support intervention? Why was it difficult or easy?
3	Ethicality	Did you have any ethical concerns regarding the intervention? If so, what were they?
4	Perceived effectiveness	In your view, how effective was the intervention in meeting your sensory support needs? Can you describe any specific ways in which the intervention has helped (or not helped) you?
5	Intervention coherence	How clear was it to you how the components of the intervention would help support your hearing and vision needs?
6	Self‐efficacy	How confident did you feel in applying the strategies or techniques from the intervention in your daily life?
7	Opportunity costs	How do you perceive the balance between the benefits of engaging in the intervention and any potential downsides?
8	General acceptability	The sensory support intervention incorporated discussion about your [or your family member's sensory support needs], training in the use of your hearing aids/spectacles, communication training and other supports. In general, what were your thoughts about the intervention, and how acceptable were each of these components of the intervention to you?

***Causal Mechanisms***

**Table:**

Capability	How did the sensory support of your hearing and vision impact your daily life? In what ways did the sensory support intervention change your understanding of your hearing or vision loss? In what ways did the sensory support intervention influence your uptake, usage and care of your [hearing aids/spectacles]? In what ways did the sensory support intervention change your interaction and communication between you and your family member/key person? ○ *Probe: knowledge, skills, decision‐making, planning*
Opportunity	Was there anything in outside factors (e.g., time, resources, etc.) that either helped or hindered your uptake of the different components of the sensory support intervention? (Probe: correct usage of sensory device, uptake of communication skills, etc.) To what extent did your family member support your uptake and usage of the different components of the sensory support intervention (e.g., correct use of sensory device, communication skills, etc.)
Motivation	How did the individual goal‐setting activity influence your usage of your hearing aids/spectacles? How did the individual goal‐setting activity benefit you? How did the sensory support intervention change your beliefs about wearing or using your hearing aids or spectacles? What impact did the sensory support intervention have on your confidence in communicating with your significant other?

***Contextual Issues***

***Preface:*** Now, I would like to explore factors that might affect the implementation of the intervention. With these next questions, I would like you to consider factors that might impact implementation from a wider perspective.

**Table:**

	Construct	Questions
1	Geographical	How might the broader physical environment or resources have influenced the uptake of the sensory support intervention?
2	Epidemiological	How might the level of someone's impairment impact the uptake of the sensory support intervention?
3	Socio‐cultural	Are there any social norms or cultural beliefs that might either support or hinder the uptake of the sensory support intervention? If yes, what are those social norms or cultural beliefs?
4	Socio‐economic	Potentially, the sensory support intervention might be user pay or a co‐pay. How might having a cost to access impact the uptake of the sensory support intervention of other people you know?
5	Ethical	Can you share your thoughts on any ethical concerns that there might be with the sensory support intervention?
6	Legal and political	Do you perceive any existing laws, regulations or political considerations that might either hinder or facilitate the uptake and adoption of this intervention? Can you tell me more? Can you identify any legal or political factors that could affect the intervention's uptake, and how would you recommend navigating these issues to ensure inclusive access and successful implementation?

**General Question:**

Based on your experience, are there any specific aspects of the intervention that you would suggest improving? This could be related to design, usability or any other factors.

Do you have any further thoughts on the sensory support intervention you would like to share with us?
